# Health workforce shortage – doing the right things or doing things right?

**DOI:** 10.3325/cmj.2022.63.107

**Published:** 2022-04

**Authors:** Aleksandar Džakula, Danko Relić, Paolo Michelutti

**Affiliations:** 1 Andrija Stampar School of Public Health, School of Medicine, University of Zagreb, Zagreb, Croatia; 2 Health Observatory Project: UP.04.2.1.06. European Social Fund, Zagreb, Croatia *adzakula@snz.hr*; 3 European Economic and Social Committee; 4 National Agency for Regional Health Services, Rome, Italy

Healthcare workforce shortage is a worldwide problem (1). Workforce shortage may be defined as not having the right number of people with the right skills in the right place at the right time, to provide the right services to the right people (2). In this regard, the trends are worrisome, and the situation is getting worse. The consequences are also very consistent – limited care health services and limited quality of health care (1). In short, there is an imbalance between need and supply. The solution: as health care needs increase worldwide, the “production” of personnel must be increased! But is it really that simple? Are the problem and the solution so reciprocal and directly linked?

Healthcare is one of the most complex public systems of the modern era, under constant intense pressure from changes in biomedicine and society (3). The coronavirus disease 2019 (COVID-19) pandemic has once again shown how the health status of the population is reflected in all segments of society. It has also shown the importance of comprehensive and sustainable human resources management in health care (4). However, the sphere of decision-making and long-term planning continues to be dominated by the problem of health care workforce shortages and their immediate consequences – limited availability and declining quality of care, as well as health care workforce burnout. While this approach draws attention to the problem of health care workforce, it is questionable whether it can contribute to the solution. Is the “problem-oriented” approach correct and sufficient, or is it perhaps incapable of creating comprehensive and sustainable solutions (5)? Is there a risk that “solutions” for health care professionals will create new problems and make the situation more difficult in the long run?

## Workforce shortage – multiple perspectives

Shortage of health care professionals has been discussed at various levels – in conversations with citizens, in debates with experts, and in the political arena. In this multidimensional world, the health care workforce problem has a complex meaning: are we looking for a solution to citizens' health needs or to their expressed demands? Are we responding to the expectations of the health system – its organizational model, its way of working, and the goals set by managers – or to the expectations of the health workers themselves? Whichever is true, we must accept that the problem has multiple dimensions, but also that the existing perspectives are mostly problem-oriented (6).

Whether and what kind of care is provided to the most disadvantaged sectors of society is determined by these critically important factors: who enters the workforce (its composition); how they are educated and trained; how they are distributed geographically and by specialty; which patients and communities are served; how their practice is oriented; and the working conditions of the entire health care workforce – including home health care workers, support staff, allied health professionals, public health, physicians, nurses, and many others (7)

In this perspective, the emphasis is on the problem and its consequences, that is, primarily on the present and the past. There is insufficient space for a full solution that includes clear visions and policy options. Narrowing the range of solutions may lead to new constraints. Therefore, it is important to apply one of the most important rules of change management – change the perspective (8). Or, as Albert Einstein put it: “We cannot solve our problems with the same thinking we used when we created them.“

## What is good for the system is not necessarily good for the citizens

Efforts in planning and managing health care workforce have clear objectives: ensuring sufficient numbers of health professionals and recruiting professionals who will ensure the effectiveness, efficiency, and quality of care and who will support the system development. Achieving such complex goals at the 80% level would certainly be a success. But even then, what about the remaining 20% of unmet goals? How are they reflected in reality – as lower incomes, lack of profits, poor indicators of outcomes of services and care provided, or inaccessible health care for some citizens? In short, a successful management of health care workforce may mean increasing inequality and new problems for some citizens. This situation is exacerbated by pressures to introduce even more intensive business models, to prioritize economic evaluations, and ultimately to make a profit. For all these reasons, health care workforce planning must be based on the principle of fairness.

## What is good for the system is not necessarily good for the health worker

The health care system thrives on the interaction of actors in the system and on business processes that are often highly regulated – medically, legally, and economically (9). However, day-to-day activities are driven by the specific needs of each patient. To put it simply, a complex health care system must ensure the highest possible standardization of clinical and business processes, but also address the specific needs of each individual patient. In practice, this is more than a challenge, and it is this duality that makes the work of health care professionals special. This is why they often deserve special status and benefits.

But do these recognitions correspond to what medical professionals need? Intensive work, stress, and the need for long-term planning of training and professional development in the medical field – these are the problems that plague health professionals (10,11). Moreover, long-term training and 24/7/365 work directly affect private life. *Vice versa* is also true – private circumstances and background considerably affect professional development and work. In addition to all this, the COVID-19 pandemic has highlighted several other challenges, most notably burnout and the mental health of health care workers (12). Are we taking them sufficiently into account when planning the health care workforce? Is ignoring the needs of health professionals a risk to planning? Or does better exploring this dimension allow us to translate it into more precise actions in health care workforce management?

The health care workforce planning is a complex challenge for the whole society. It is important to clearly define and consider the needs of citizens and health workers, as opposed to simply fulfilling the gaps in the system. In setting the goals, the priorities should be quality primary care and equitable access to care. Workforce policies and requests for greater efficiency must not deepen the inequities for some patients or deprived population groups. When planning and managing processes, we have to identify all the needs of the professionals – from working conditions, material rights, opportunities for promotion, to the creation of a sustainable balance between private and professional life.

On the positive side, these priorities are already being recognized in the main EU documents and many policy documents globally (1,13,14). However, this is not reflected in the approach and interventions used, as they still do not promise positive and sustainable change. Therefore, the question remains: are we still trying to solve our problems with the same thinking we used when we created them?
